# Temporal Changes in Extended Spectrum β-Lactamase Producing Organisms in Acute Care Surgery

**DOI:** 10.7759/cureus.32190

**Published:** 2022-12-04

**Authors:** Arslan Zahid, Hina Aslam, Aayanoor Zahid, Iftikhar Ahmed, Saba Aslam, Zunera Jahanzeb, Atta-Ul-Aleem Khalid

**Affiliations:** 1 Surgery, Hazrat Bari Imam Sarkar (HBS) Medical and Dental College, Islamabad, PAK; 2 Internal Medicine, Hazrat Bari Imam Sarkar (HBS) Medical and Dental College, Islamabad, PAK; 3 Various, Mayo Hospital, Lahore, PAK; 4 Emergency Department, District Headquarters Hospital, Chakwal, PAK; 5 Surgery, Armed Forces Hospital, Muscat, OMN

**Keywords:** antibiotic stewardship program, surgical wound infection, gram-negative bacterial infections, antibiotic resistance (abr), esbl+

## Abstract

Background: Extended-spectrum β-lactamase (ESBL) producing organisms are resistant to a wide range of broad-spectrum antibiotics, and their emergence is a significant driving force of antibiotic resistance. Most South-Asian countries have become hotspots for antibiotic resistance, so specifics of ESBL data are critical to tackling antibiotic resistance. We present the temporal changes in ESBL-producing organisms cultured in our tertiary care referral centre.

Methods: Over a year, a historical cohort analysis was carried out at our tertiary care referral centre in Southeast Asia. Samples from patients presenting with acute surgical conditions were sent for culture and sensitivity. The phenotype of all specimens was checked using the combination disc method. Antimicrobial susceptibilities to various antibiotics were also checked as per CLSI (Clinical and Laboratory Standard Institute) guidelines.

Results: Specimens from 170 patients were analysed. The mean age was 44.73±19.89 years, and there was a female predominance of 62%. The most common organisms were *Escherichia coli* (70%), *Klebsiella* *pneumoniae* (18%), and *Pseudomonas aeruginosa* (16%). The percentage of ESBL-producing organisms was 54.7%, which is significantly higher than in previous reports. Widespread resistance was found against commonly used antibiotics, including co-amoxiclav (81.9%), ceftriaxone (75%), ciprofloxacin (47%), and levofloxacin (35.7%). Sensitivities to combination antibiotics like piperacillin-tazobactam (79.2% sensitive), cefoperazone-sulbactam (84.3% sensitive), and imipenem-cilastatin (91.1% sensitive) were also noted to be falling.

Conclusion: The incidence of ESBL-producing organisms continues to increase at an alarming rate, which mandates strict antibiotic stewardship and amendments to local guidelines.

## Introduction

Antimicrobial resistance is one of the forefronts of battle in current medical practice. Increasing resistance to common as well as uncommon antimicrobial agents is alarming, and this problem has been declared a global threat to public health [1]. South Asia is considered a hotspot for emerging strains of resistant organisms, with multiple factors contributing to the issue. Many factors contribute to the problem in this region, including but not limited to the lack of antimicrobial stewardship, unrestricted prescription of broad-spectrum antibiotics, over-the-counter availability of antibiotics without a prescription, and patient compliance [2].

Gram-negative bacteria (GNB), particularly *Escherichia coli*, *Klebsiella pneumoniae*, and *Pseudomonas aeruginosa*, were traditionally treated with penicillins, cephalosporins, and fluoroquinolones. After the acquisition of β-lactamases and extended-spectrum β-lactamases (ESBL) by these microbes, clinicians have been struggling to treat them even with broad-spectrum antibiotics like third-generation cephalosporins. This is a cause for concern as the choice of antibiotics to treat these multi-drug-resistant organisms is becoming very limited, and there might not be any viable antibiotics left if the problem is not addressed emergently [3].

Whereas β-lactamases were only resistant to Penicillins and first- and second-generation cephalosporins, mutations in β-lactamases resulted in the evolution of ESBL, which provides resistance to third-generation cephalosporins as well as monobactams [4]. The detection of ESBL production is being seen frequently in current clinical practice [5]. Abrar et al. published a meta-analysis in 2018 on the prevalence of ESBL-producing Enterobacteriaceae in Pakistan and included studies done from 2000 to 2015 [6]. Their results showed that the proportion of prevalence of ESBL production was 0.40 (95% CI: 0.37-0.47). Their results were quite concerning, as the ESBL burden in Southeast Asia is alarmingly high as compared to western countries like the United Kingdom, which reported ESBL production in 10% (proportion: 0.10) of the isolates in 2009 [7]. This is a major cause for concern, as the disparity between the two proportions indicates the difference made by active surveillance and good antimicrobial stewardship policies. Other studies from different regions of Pakistan have also shown an increase in the prevalence of ESBL-producing organisms [2,8-10]. In this study, we present our data on the prevalence of ESBL-producing organisms from a tertiary care referral in Southeast Asia.

## Materials and methods

A historical cohort analysis was carried out. Ethical approval was obtained from the local ethics committee (Appl# EC, 02/29/03/2021). Specimens sent for culture and sensitivity underwent Gram staining after initial inoculation in blood and MacConkey agar for wound and pus specimens, and cystine lactose electrolyte-deficient (CLED) agar for urine specimens. All Gram-negative organisms had their antibiotic susceptibilities checked by the Kirby-Bauer disc diffusion method on nutrient agar with standard antimicrobial discs as per Clinical and Laboratory Standard Institute (CLSI) guidelines. A total of 170 cultures were included from January 2019 to June 2020. All cultured organisms were checked for antimicrobial susceptibility against Penicillins, first-, second-, and third-generation cephalosporins, fluoroquinolones, carbapenems, aminoglycosides, and tetracyclines. Representative drugs were used from the above classes in all cultures. ESBL production was confirmed by using the combination disc method (ceftriaxone alone versus ceftriaxone plus clavulanic acid and/or cefotaxime alone versus cefotaxime and clavulanic acid). Organisms were phenotypically considered ESBL-producing when the zone of inhibition was ≥5 mm larger around the combination discs as compared to antibiotic discs. Data analysis was performed using IBM SPSS version 22. A chi-square test was used to compare frequencies, and an unpaired student t-test was used to compare means.

## Results

**Figure 1:**
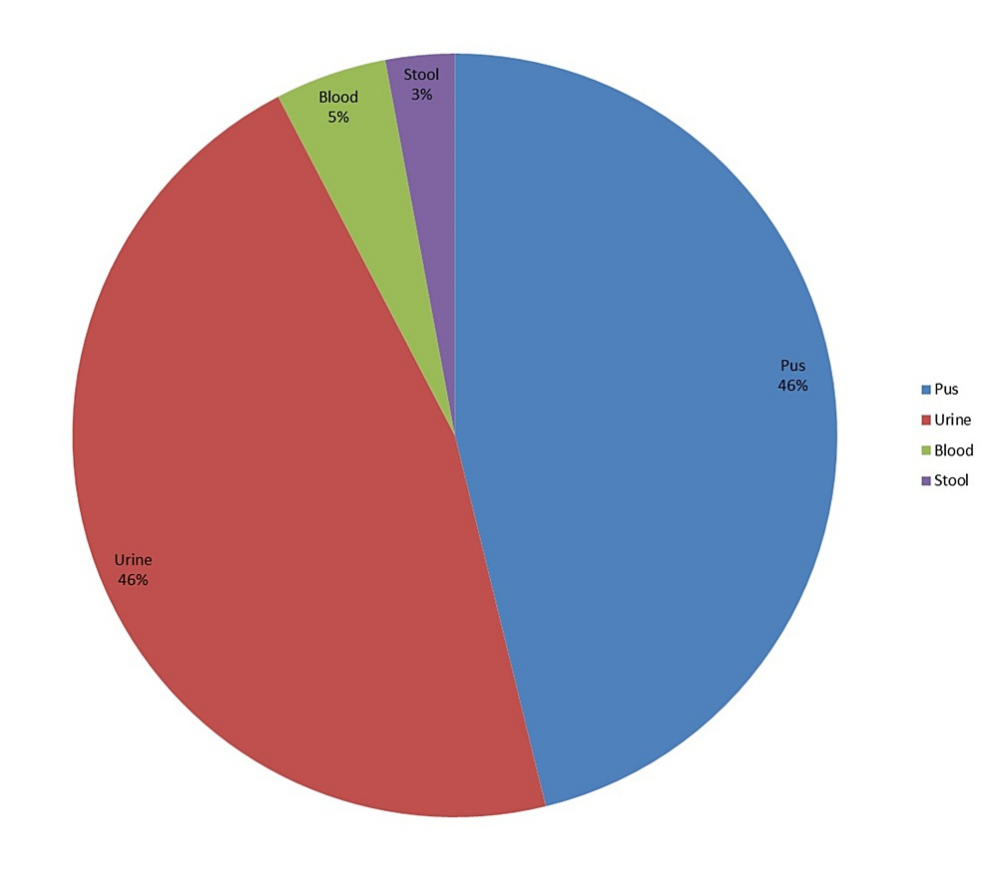
Specimens used to culture isolates in our study

**Figure 2:**
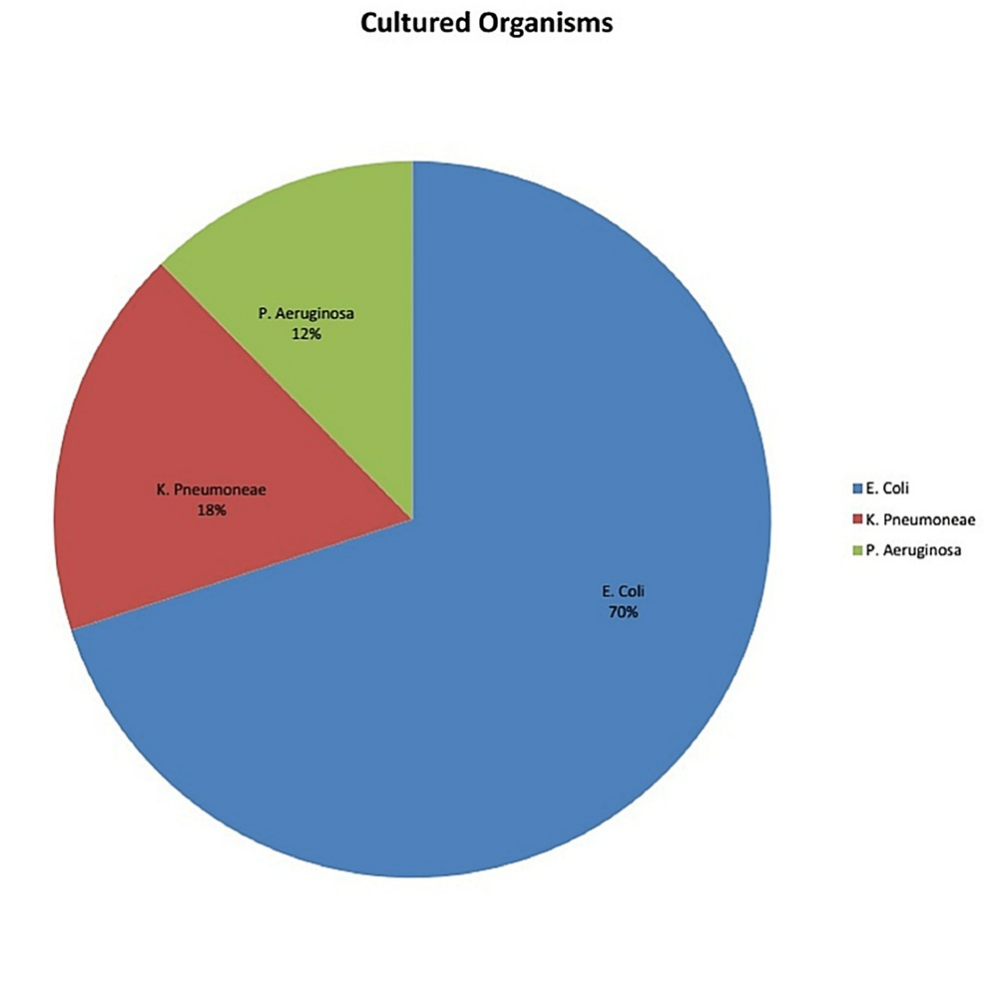
Distribution of Gram-negative bacteria isolated from cultures

**Figure 3:**
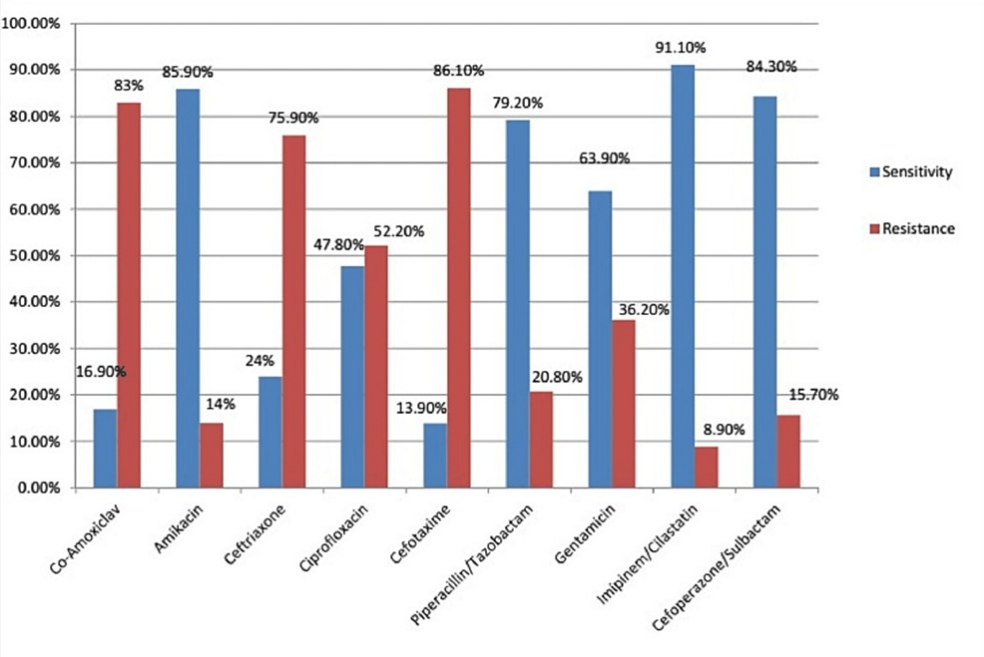
Bar graph demonstrating the levels of resistance and sensitivity of different antibiotics

Specimens from 170 patients were included in the study. There was a female predominance (62%). The mean age was 44.73±19.89 years. The distribution of microbes isolated from these cultures is elaborated in Figure 1. Figure 2 shows the relative incidence of the organisms cultured in our study. The percentage of ESBL-producing organisms was found to be 54.7% (n=93). Figure 3 demonstrates the susceptibilities of different antibiotics. A chi-square test was performed to see whether gender was related to ESBL status, but the results were insignificant (p=0.191), unpaired A Students t-test was applied to check if there was a relationship between ESBL status and the age of the patient; this was also insignificant (p=0.546, Students t-test). Table 1 shows the frequency of ESBL producers and non-producers according to the type of organism and specimen used for culture.

**Table 1:** The proportion of extended-spectrum β-lactamase-producing Gram-negative bacteria compared to non-extended-spectrum β-lactamase producing Gram-negative bacteria

	ESBL negative (n=77)	ESBL positive (n=93)
Organism		
*E. coli*	49 (41.2%)	70 (58.8%)
*K. pneumoniae*	11 (36.7%)	19 (63.3%)
*P. aeruginosa*	17 (81%)	4 (19%)
The specimen used for culture		
Pus	41 (52.6%)	37 (47.4%)
Urine	29 (37.2%)	49 (62.8%)
Blood	7 (87.5%)	1 (12.5%)
Stool	Nil	5 (100%)
Semen	Nil	1 (100%)

Our study showed high levels of resistance to common antibiotics like co-amoxiclav (81.9% resistant), ciprofloxacin (47% resistant), levofloxacin (35.7% resistant), and ceftriaxone (75% resistant). Isolates were found to be sensitive to combination antibiotics like piperacillin-tazobactam (79.2% sensitive), cefoperazone-sulbactam (84.3% sensitive), and imipenem-cilastatin (91.1% sensitive), while aminoglycosides like amikacin and gentamicin also showed a reasonable sensitivity rate of 85.9% and 64%, respectively.

Another interesting finding in our study was that the number of resistant organisms to co-amoxiclav, ciprofloxacin, levofloxacin, tazocin, and gentamicin was significantly higher for ESBL-producing organisms as compared to non-ESBL-producing organisms. This is illustrated in Table 2.

**Table 2:** Comparison of sensitivity and resistance of various antibiotics between extended-spectrum β-lactamase-producing and non-extended-spectrum β-lactamase-producing Gram-negative bacteria

Antibiotic		ESBL status		p-value
		ESBL producers	Non-ESBL producers	
Amikacin	Resistant	12.1%	4.9%	0.329
	Sensitive	87.8%	95%	
Co-amoxiclav	Resistant	89.4%	65%	0.02
	Sensitive	10.5%	34.6%	
Ciprofloxacin	Resistant	60.8%	30%	<0.001
	Sensitive	39.1%	70%	
Tazocin	Resistant	17%	5.5%	0.02
	Sensitive	82.9%	94.5%	
Gentamicin	Resistant	37.8%	17.7%	0.021
	Sensitive	62.2%	82.2%	
Co-trimoxazole	Resistant	82.8%	65.8%	0.041
	Sensitive	17.1%	34.1%	
Levofloxacin	Resistant	44.4%	26.4%	0.047
	Sensitive	55.5%	73.5%	

## Discussion

ESBL production by Enterobacteriaceae renders them resistant to even third-generation cephalosporins. Over time, there has been a rise in the number of ESBL-producing Enterobacteriaceae because of antibiotic resistance, as our study demonstrated that more than half of the isolates in our study were ESBL-producing. Our results show a significant rise in the multidrug resistance of ESBL-producing GNBs, with particularly high resistance rates to commonly used antibiotics including co-amoxiclav (89.4%), ciprofloxacin (60.8%), levofloxacin (44.4%), gentamicin (37.8%), and rising resistance rates for broad-spectrum antibiotics like piperacillin-tazobactam (17%). The proportion of ESBL-producing GNBs has also risen at an alarming rate to 54.7%.

Antimicrobial drug resistance is an alarming concern in current medical practice. This alarming issue, especially in regions with poor antimicrobial stewardship such as South Asia, limits the efficacy of stronger antibiotics. The use of antimicrobials is one of the contributing factors to the development of antimicrobial resistance, and some authors have suggested that over-prescription of antimicrobials may be responsible for the increasing levels of antimicrobial resistance over time [11].

Comparing our results with previous studies from the same region shows that the frequency of ESBL-producing Enterobacteriaceae has increased significantly. Jamil et al. cultured 75 isolates from 240 urine samples and reported an ESBL-production frequency of 25 (33.3%) [8]. Khan et al. published a six-year study from 2002 to 2007 that included more than 15,000 K. pneumoniae isolates out of which 31% (n=5016) were found to be ESBL producers [10]. In our study, only 30 (17.6%) isolates grew K. pneumoniae, and of these isolates, 19 (63.3%) were found to be ESBL producers. Abrar et al. in their meta-analysis reported a pooled proportion of ESBL producers to be 0.40 (95% CI: 0.34-0.47), which is lower than what we found in our study [6]. They included 55 studies from 2002 to 2015. Their study showed a subtotal pooled estimated proportion of 0.33 (95% CI: 0.21-0.46), while the estimated proportion of a nearby region was 0.50 (95% CI: 0.39-0.62). All of the studies reporting data from this region were conducted during or before 2015, which puts this data at a significant time interval from our results. Considering the overall pooled estimates from previous studies, there has been a significant rise of 15% in the proportion of ESBL-producing Enterobacteriaceae.

A comparison with international reports from different parts of the world shows varying numbers regarding the prevalence of ESBL producers in different geographical regions. Flokas et al. in their meta-analysis of pediatric urinary tract infections reported a pooled prevalence of 14% (95% CI: 8-21) among included studies, with a 12% (95% CI: 2-31) estimated prevalence from Europe from five studies and an estimated prevalence of 5% (95% CI: 0-7) in the Americas from three studies [12]. Ghotaslou et al., in their study from Iran, reported the prevalence of ESBL to be 42.7% (131 out of 307 isolates) [2]. Abayneh et al., in their study from Ethiopia, reported that 23% (17 out of 74 isolates) of the isolates were ESBL producers [13]. There is a general trend towards increased ESBL-producing bacteria in Southeast Asia, as corroborated by other studies.

The susceptibilities of ESBL to various antibiotics showed an alarming level of resistance to commonly used drugs, while higher-level antibiotics such as carbapenems showed adequate levels of sensitivity when used in combination. The levels of resistance to other drugs are also on the rise, with 30% of the isolates being resistant to gentamicin. Other drugs like Linezolid have been used injudiciously in recent times, which has resulted in an alarming rate of resistance against this antibiotic; our data showed that 63% of the tested isolates were resistant to Linezolid. This is an alarming finding as Pakistan has already failed to achieve the targets set by the WHO’s 2017 mission report on antimicrobial resistance as per the global action plan of the World Health Assembly 2015.

ESBL-producing organisms can be tackled by judicious use of broad-spectrum antibiotics, increasing the rate of cultures, and sparing antibiotics that have high levels of sensitivity. Carbapenem-sparing strategies are being practiced in some parts of the world to preserve some antibiotics as a last line of defence against ESBL-producing organisms [12]. Emergent steps need to be undertaken to tackle the menace of antibiotic resistance. The previously identified factors of polypharmacy, a large number of registered products, irrational prescriptions, over-the-counter availability without prescription, quackery, and a shocking lack of surveillance systems need to be rectified on an urgent basis. Rigorous antimicrobial stewardship policy and education need to be instituted if we are to stand a chance against antibiotic resistance in the future.

## Conclusions

Our study showed a significant increase in the frequency of ESBL-producing organisms as compared to previous studies. In our study, more than half of the cultured organisms were found to be ESBL-producing with high levels of resistance to commonly used antibiotics. Emergent steps are needed to contain the rapidly rising resistance levels among bacteria, particularly in South Asia. These steps should include strict antimicrobial stewardship, judicious prescription of broad-spectrum antibiotics, and physician training to improve the practice of culturing organisms and the prescription of targeted antibiotics after cultures. These steps must be undertaken immediately to preserve the efficacy of antibiotics and tackle the menace of antibiotic resistance.
